# Behaviors and practices of incarcerated women towards menstrual hygiene in a large urban prison in Uganda: a phenomenological qualitative study

**DOI:** 10.1186/s12905-023-02462-5

**Published:** 2023-06-27

**Authors:** Margaret Nabiryo, Miriam Ondia, Jonathan Izudi

**Affiliations:** 1 Institute of Public Health and Management, Clarke International University, Kampala, Uganda; 2 Department of Community Health, Faculty of Medicine, Mbarara University of Science and Technology, Mbarara, Uganda; 3 Infectious Diseases Institute (IDI), Makerere University College of Health Sciences, Kampala, Uganda

**Keywords:** Behaviors and practices, Menstrual hygiene management, Incarcerated women

## Abstract

**Background:**

Deplorable and unconducive conditions in prisons present serious challenges to menstrual hygiene management. However, little is known about menstrual hygiene among incarcerated women in Uganda. Our study explored the behaviors and practices of incarcerated women regarding menstrual hygiene management in a large government prison in Uganda. In addition, we explored the barriers to menstrual hygiene management in this population.

**Methods:**

In this phenomenological qualitative study, we conducted in-depth interviews with incarcerated women aged 20–49 years and key informant interviews with female prison officers (wardresses) at Luzira Prison in Kampala, Uganda. The data were analyzed using content analysis and the findings were presented using themes/sub-themes along with participant quotes.

**Results:**

We interviewed 15 incarcerated women aged 20–49 years (mean age, 29.5 ± 8.7 years) and five key informants aged 30–50 years (mean, 42.6 ± 4.9) about menstrual hygiene behaviors and practices, including barriers to menstrual hygiene. Five sub-themes emerged concerning behaviors and practices of menstrual hygiene among incarcerated women. Findings reveal the behaviors and practices of menstrual hygiene management were characterized by infrequent change of menstrual pads, lack of privacy during menstrual hygiene practices, use of poor-quality menstrual hygiene materials, and improper disposal of used sanitary products. However, bathing with soap and water during menstruation was frequent and non-restricted. Three sub-themes emerged as barriers to menstrual hygiene practices, largely at the institutional level, and they included unhygienic sanitary facilities, unreliable access to clean water, and insufficient sanitary products.

**Conclusions:**

Behaviors and practices of incarcerated women fall short of desired standards and they face several barriers to practicing menstrual hygiene. The prison authorities should provide sufficient sanitary products like pads, and knickers including soap, construct more sanitary facilities, educate about the safe disposal of used sanitary products, and provide sufficient clean water to promote good menstrual hygiene management among incarcerated women.

## Background

Estimates suggest that more than 800 million women and adolescent girls menstruate every day [1], and of these, at least 714,000 are women and girls in prison [2]. In low and middle-income countries, the practice of menstrual hygiene among women in prison is challenging due to a lack of basic menstrual hygiene management products and difficult access to sanitary facilities [3]. Evidence indicates that slightly over six in 10 women in prison lack sufficient sanitary facilities for menstrual hygiene management [4]. The majority of prisons in sub-Sahara Africa are overcrowded, unclean, and not favorable to promoting the practice of menstrual hygiene management among incarcerated women [5].

Uganda has 74,444 people in prison as of January 12, 2023, with 4.6% being women [2]. The World Prison Brief Report [2] indicates that the carrying capacity of the prison system in Uganda is more than 371%. Currently, female prisoners are held in more than 110 prisons, but just 13 of them have facilities for managing menstrual hygiene. In rural settings, most of the prisons have no separate facilities for women, resulting in shared facilities such as toilets between men and women [2]. A previous study [6] has shown that the menstrual challenges among incarcerated women include inadequacies in sanitary facilities such as toilets, insufficient disposable sanitary pads, and forced use of reusable clothes or hand-made sanitary pads that are made from cotton wool and scrap fabric.

The conditions of prisons in Uganda are deplorable and unconducive [7], raising serious concerns about the sexual and reproductive health of incarcerated women, particularly behaviors and practices regarding menstrual hygiene management. Currently, there is insufficient information about menstrual hygiene management among incarcerated women in Uganda. Without information about the behaviors and practices of incarnated women regarding menstrual hygiene management, evidence-informed decisions regarding the planning and improvement of menstrual hygiene facilities in prison settings might prove difficult to achieve. Therefore, this study explored the behaviors and practices of incarcerated women regarding menstrual hygiene management in Kampala, Uganda. We also described the barriers to menstrual hygiene management among incarcerated women.

## Methods and materials

### Study design, setting, and population

We conducted a phenomenological qualitative study at Luzira Prison, the largest public prison established in 1927 in Kampala. A qualitative study design was deemed fit since little information exists regarding menstrual hygiene management in a prison setting so the use of the design enabled an in-depth exploration and understanding. There are approximately 3,300 inmates in this prison and 306 are women. We studied incarcerated women aged 20–49 years. Eligible participants were those incarcerated for ≥ 1 month and who had experienced a menstrual period in the past month. The length of prison stay was established from existing records. Of 150 women approached for participation in the study, 100 declined to participate for reasons: (1) uncomfortable and shameful to share personal menstrual hygiene experiences; (2) fear to provide information on menstrual hygiene in a prison setting; and, 4) being busy with prison scheduled chores. In addition to the incarcerated women, we studied female prison officers (wardresses) who had been employed at the study site for ≥ 5 years. We found five eligible prison officers, and all were interviewed.

Regarding sampling, all the female prison officers were purposively sampled in the study. The sampling of the 50 incarcerated women who had accepted to participate in the study depended on a simple random sampling approach which was easy and quick because the sampling frame was known and the total population of incarcerated women was relatively small (n = 306), at the time of the study. Here, we assigned unique codes that ranged from 1 to 50 to the incarcerated women who had consented to participate in the study in a Microsoft Excel sheet. We used the codes to randomly select 40 eligible women for the interviews and the interviews were done until the desired sample size was reached.

### Study variables

Our study was guided by several topics that focused on access to sanitary products for menstrual hygiene management, types of sanitary pads provided by the prison authority, source and supply of sanitary products, quantity and quality of sanitary products, and affordability of sanitary products. The topics regarding menstrual hygiene behaviors and practices included the frequency of changing used sanitary pads, disposal of used sanitary pads, facilities for bathing, frequency of bathing, and privacy during menstruation. Furthermore, we focused on institutional support for menstrual hygiene management.

Here, we interviewed the women about the availability of sanitary facilities, accessibility to sanitary products, the cleanliness of the sanitary facilities, and the disposal of used sanitary pads. Female prison officers were interviewed regarding accommodation facilities within the prison, support to incarcerated women during menstruation, and existing prison services to support menstrual hygiene management.

### Data collection

Data were collected using an in-depth interview (IDI) guide for incarcerated women and a key informant interview (KII) guide for female prisoner officers. In developing the IDI and KII guides, we first outlined the areas of knowledge relevant to answering the research question, developed main questions within each of the areas, revised the questions to ensure they are simple and easy to understand, and constructed the main questions using “How” rather than “Why” along with probing questions, all in chronological order. We placed easy questions at the start and difficult questions at the end of the guides. The interviews were conducted by two interviewers (MN and JG), both are public health specialists with a Master of Public Health (MPH) degree. MN asked the questions while JG took field notes and probed whenever necessary. The audio recording was not possible because recording devices were not allowed within the prison setting. The interviews were conducted in a quiet, designated room, lasted 40–50 min for the IDIs and 30–40 min for the KIIs, on average.

### Data analysis

We did not compute the sample size using a formula but relied on the saturation principle as reported elsewhere [8]. Our a priori sample size was 50 participants (40 incarcerated women and 10 prison officers). However, we interviewed 15 incarcerated women and 5 female prison officers (Wardresses), overall 20 participants to attain saturation. We performed content analysis and adopted an inductive approach. We cleaned the verbatim field notes within 6–12 h of the data collection. MN and JG read a few of the transcripts several times to gain familiarity and then developed a preliminary codebook that guided the coding of the remaining transcripts. However, both MG and JG allowed new codes to emerge whenever deemed appropriate. The coding was independently performed by MN and JG to prevent subject bias.

Once the coding was completed, the preliminary codebook was revised to form a final codebook which was verified by a third analyst, MO (female public health researcher with training and experience in qualitative research). We presented the themes/sub-themes along with the participant quotes.

### Ethical considerations

Clarke International University Research Ethics Committee reviewed and approved the study (CIU-REC/0139). Administrative clearance was obtained from the Office of the Commissioner General of Prisons in Uganda and was presented to the Officer-In-Charge of Luzira Prison. We established a schedule for data collection for both the incarcerated women and wardresses. All participants provided informed consent in writing or by thumbprint. Before acquiring informed consent, we explained the purpose of the study, the selection criteria, the study benefits and potential harms, and the withdrawal process in case the participants wanted to opt out of the study. We assured the participants of the privacy and confidentiality of the information. For example, for each participant, we used anonymous codes during the data collection. The use of unique codes ensured confidentiality during the data analysis and presentation of the findings. Privacy was ensured by conducting the interviews in a quiet room within the prison setting accessible to just the research team.

## Results

Data were collected from 20 participants (15 incarcerated women and 5 prison wardresses). Table 1 presents the demographic characteristics of the participants and shows that the majority of the incarcerated women were aged 20–29 years (53.3%) and the overall mean age was 29.47 years. Concerning the key informants (Prison Wardresses), the majority were aged 30–40 years and the mean average age was 42.6 years (SD = 4.9). Three of the key informants had 6–10 years of work experience.

**Table 1:** Characteristics of respondents

Variable	Level	Frequency	Percentage
Incarcerated women (n = 15)			
Age (years)	20–29	8	53.3
	30–39	5	33.3
	40–49	2	13.3
	Mean (SD)	29.5(8.7)	
Duration of imprisonment (months)	1–30	10	66.6
	31–60	4	26.7
	61–90	1	6.7
	Mean (SD)	26.4	
**Key informants or prison wardresses (n = 5)**			
Age (years)	30–40	3	60
	40–50	2	40
	Mean (SD)	42.6(4.9)	
Work experience (years)	≥5		
	6–10	3	60
	11–15	2	40
	mean (SD)	10 (2.5)	

Note: SD: Standard deviation

## Themes

Table 2 summarizes the study findings under two major themes. The first theme (Theme 1) presents the behavior and practices of menstrual hygiene and consists of five sub-themes, while the second theme (Theme 2) shows the barriers to menstrual hygiene practices and it consists of three sub-themes.

**Table 2:** Emergent themes and sub-themes

Theme	Sub-themes
Behaviors and practices of menstrual hygiene	Infrequent changing of sanitary pads.
	Poor quality menstrual hygiene materials
	Lack of privacy during menstruation hygiene management
	Improper disposal of used sanitary products
	Frequent bathing with soap and water during menstruation
Barriers to menstrual hygiene practices	Unhygienic sanitary facilities
	Unreliable access to clean water
	Insufficient sanitary products

### Theme 1: behaviors and practices of menstrual hygiene

Under this theme, five sub-themes emerged among the incarcerated women, and we present these sub-themes along with the participant quotes.

#### Infrequent changing of sanitary pads

Frequent changing of used sanitary pads was reported by certain participants, but it was limited due to inadequacies in sanitary pads. The majority of the participants needed to change their sanitary pads frequently due to heavy menstrual flow but could not do so as they had to save a few sanitary pads for the next day.

*“Some change several times [frequent change of sanitary pads]. However, some are dirty and do not bother themselves to change regularly. But, some do not change regularly to save pads for other days.”* (LP3, 50 years).

*“Actually, some months when am bleeding too much, I can change even 6 times a day, but usually I change only 3 times a day.”* (LP10, 34 years).

#### Poor quality menstrual hygiene materials

Incarcerated women mentioned having used pieces of cloth from their prison uniforms as an alternative menstrual absorbent whenever they lacked sanitary pads.

*“….. If one’s pads are over, one can request from a friend. However, if the friend does not have, one can use a piece of cloth. It is common for many of us here to cut a piece of cloth from our uniforms. But, it fills fast and stains our uniforms”* (LP11, 25 years).

*“Some feather pads are duplicates, these can itch and easily tear. They cause sores. And some expire before we use.”* (LP14, 47 years).

#### Lack of privacy during menstrual hygiene management

Privacy is important during menstrual hygiene management but it was difficult since the participants shared the existing bathing shelters with their peers.

*“Being in a congested place as a lady during menstrual periods is inconveniencing, and most women need their privacy. Each woman experiences changes during periods [menstruation periods] and some women do not want to be with others. But here in the prison, the inmates have to bear with each other because we do not have private rooms for them.”* (PO3, 50 years).

The participants equally acknowledged that a lack of privacy during menstrual hygiene management was a problem. However, they indicated that it was the only option.

*“There is no problem when another inmate sees me when I am changing a pad, bathing, or urinating because there is no privacy here. If another inmate is coming in to use the toilet, I just continue to do what I was doing. The doors do not have locks.”* (LP4, 28 years)

*“It is rare to be alone in the toilet, when I enter the toilet, there are other people who have come to bath, urinate or change their pads, though usually no prison officials ever bother to enter in our toilets.“* (LP6, 31 years).

#### Improper disposal of used sanitary products

The disposal of used sanitary pads was problematic in the prison setting. Certain participants reported the use of buckets for the disposal of used sanitary pads.

*“Some do not wrap the soiled sanitary pad and they just dispose of it in the bucket bare as it is. And some are ignorant of hygiene practices, so they just throw the soiled pad in the bucket without wrapping.” (LP3, 50 years).*

*“There are others who do not even know what to do with their used pads. You find someone putting their used pads in flower gardens, for those who do not know how to discard their pads.”* (PO1, 48 years).

#### Frequent bathing with soap and water during menstruation

The majority of the participants reported bathing 2–3 times a day during menstruation, with water and soap and without restrictions. Nonetheless, it was reported that the bathing facilities namely water, soap, and space were inadequate. The participants mentioned that they struggled with the limited bathing space, including water and soap.

*“I bath twice a day at 6 am and 9 pm, and we use the toilets as bathrooms.”* (LP1, 21 years).

*“I bath three times a day, usually bath at 7 am because the people are many after 8 am, then also in the afternoon and evening.“* (LP11, 25 years).

*“We get a quarter of a bar of soap from O.C [Officer In-charge of Prisons] and one has to use it to bath and wash knickers. So, it gets finished in one week.”* (LP5, 23 years).

### Theme 2: barriers to menstrual hygiene management

Several barriers to menstrual hygiene management practices emerged, largely at an institutional than a personal level. In the subsequent paragraphs, we present and describe these barriers.

#### Unhygienic sanitary facilities

Participants described the sanitary facilities as being unhygienic, with one of the participants reporting that the toilets were never cleaned even though cleaning support systems were available.

*“The toilets and pit latrines are not clean enough, but we have a floor rug and liquid soap for each one of us to use for cleaning the floor after bathing.”* (LP8, 21 years).

*“The toilet is dirty and also because there is no water in our ward. If the tap water stops flowing, we suffer a lot.”* (LP11, 25 years).

#### Unreliable access to clean water

Access to clean and ample water is important to promote the practice of menstrual hygiene. Although a piped water system was available onsite, most times, water was either not available, not sufficient, or unreliable so the prison authority had to search for alternative sources of water from the nearby river or lake. It emerged that the water from these sources was most times dirty and unsafe.

*“We have tap water supply in the toilet……...but we can spend 3 days without tap water; they bring the water from the river which is white and smells.”* (LP13, 30 years).

*“The inmates have water within their wards and others can also go and fetch from the taps outside their wards.”* (PO1, 48 years).

#### Insufficient sanitary products

The participants reported being provided with disposable sanitary pads by the prison authority. However, it was stated that the sanitary pads were inadequate compared to the menstrual demands since a packet of the sanitary pad was just provided.

*“…the prison service gives me one packet with eight pieces, yet I spend 9 days in my periods. So, I have to borrow and repay when I am given by my visitors.”* (LP7, 30 years).

*“Some of these inmates have a heavy flow and so the one packet of sanitary pads given is not enough. On the other hand, we have so many female inmates, and so they have to share the little number of sanitary pads provided. And then, there are times, it is not a guarantee that they give them sanitary pads.”* (PO4, 43 years).

*” If one’s pads are over, one can request from a friend. However, if the friend does not have, one can use a piece of cloth. It is common for many of us here to cut a piece of cloth from our uniforms.”* (LP9, 25 years).

*“… You have to beg for pads when you are new. When I came, I saw some inmates using a mattress. They would cut a sizable piece and use it as a pad.”* (LP15, 20 years).

## Discussion

Our study described the behaviors and practices of incarcerated women regarding menstrual hygiene management at Luzira Prison, the largest government prison in Kampala, Uganda, as well as the barriers to menstrual hygiene management. We found the behaviors and practices of menstrual hygiene management were characterized by infrequent change of menstrual pads, use of poor-quality menstrual hygiene materials, a lack of privacy during the practice of menstrual hygiene, and improper disposal of used sanitary products. However, bathing with soap and water during menstruation was frequent and non-restricted. The barriers to menstrual hygiene management included unhygienic sanitary facilities, unreliable access to clean water, and insufficient sanitary products. Overall, the behavior and practices of menstrual hygiene management among incarcerated women did not conform to the desired menstrual hygiene standards. The negative behaviors and practices included a less frequent change of sanitary pads, a lack of privacy, and improper disposal of used sanitary pads. Appropriate menstrual hygiene practices recommend changing pads every 2–6 h [9] to prevent infections, spills, and unpleasant odors. The inadequacies we present in this study have been highlighted in several past studies [7, 10–13] and they present a health risk such as infection and psychological problems like stress, depression, and anxiety.

The unhygienic disposal of used sanitary items that we report is documented in previous studies in Nepal [14] and southern Senegal, Zimbabwe, South Africa, Malawi, and Zambia [5]. Van Hout et al. (2018) reported that no measures exist for the disposal of used sanitary pads in prison settings, which is consistent with our findings.

The problem of lack of privacy during menstrual hygiene practices is equally reported elsewhere [10]. A lack of privacy during menstrual hygiene practice leads to a loss of dignity, respect, and self-esteem among incarcerated women. Our findings highlight real-world challenges in promoting menstrual hygiene management practices among incarcerated women in this setting. The findings emphasize a need for the prison health authorities to address the prevailing menstrual hygiene management constraints by improving the supply of sanitary pads and subsequently their disposal.

Our finding of frequent bathing with soap and water, on average 2–3 times per day and without restrictions is impressive because frequent bathing is recommended during menstruation [6]. This finding is consistent with one study conducted in Ethiopia and Malawi that report female inmates have access to soap and water to support bathing and the washing of clothes, including reusable menstrual absorbent clothes [5]. In South Africa, a study reports that incarcerated women are provided with soap [10], which agrees with our findings. Frequent bathing will go a long way in maintaining personal hygiene during menstruation, improving comfort, and preventing reproductive tract infections among incarcerated women. The lack of restrictions regarding the frequency of bathing during menstruation might be a result of the prioritization of menstrual hygiene over the years.

We found incarcerated women periodically received free menstrual hygiene materials although it was reported the materials could not adequately absorb menstrual blood, with some women reporting having experienced blood-stained clothes. Our finding conforms to a previous study that reports incarcerated women are mostly provided with poor-quality menstrual absorbents that have inadequate absorption capacity for menstrual flow [15]. Our findings imply a need to adhere to the World Health Organization guideline of providing incarcerated women with essential sanitary products that are of good quality at no cost [15]. Failure to do so will compromise the maintenance of good menstrual hygiene practices leading to infection, shame, and discomfort among others. Our data show that incarcerated women face several barriers to practicing good menstrual hygiene. For example, an unhygienic prison setting, insufficient and unreliable access to clean water, and inadequate provision of sanitary products like pads among others. Our findings are consistent with the results of two previous systematic reviews that report a shortage of water supply and restricted access to sanitary facilities leading to infrequent bathing during menstruation [5].

Inadequacies in sanitary facilities have been reported to compel incarcerated women to share the few available facilities [16], with more than 50 sharing a sanitary facility during menstruation [3]. In part, the insufficiencies are a result of overcrowding as has been reported earlier [9, 10]. The barriers we report in this study in general contribute to difficulties in maintaining good menstrual hygiene practices among incarcerated women and required urgent mitigation measures such as increasing menstrual hygiene facilities and decongesting the prison cells and so forth.

### Study strengths and limitations

Our study has strengths and weaknesses. Regarding the strengths, menstrual hygiene management in a prison setting is understudied. The present study is among the few studies to provide insights into this topic. Our findings hence set a benchmark for future research. Second, since this study was conducted in one of the largest government prisons in Uganda, the findings might paint a picture of menstrual hygiene practices at other government prisons in the country. Third, by interviewing female prison officers (wardresses) as key informants, our findings provide a better understanding of institutional support and challenges in promoting good menstrual hygiene practices among incarcerated women. The limitations of the study include possible data inaccuracies as audio-recoding was not permissible. The data reported in this study are from an urban prison setting, so might not exactly replicate findings from a rural prison due to systematic differences in participant characteristics. We did not collect data about the quality of the menstrual materials provided by the prison authorities to the incarcerated women. These limitations should be considered in the interpretation of the findings.

## Conclusions and recommendations

Our study shows that menstrual hygiene behavior and practices of incarcerated women at the Luzira Prison, the largest government prison, in Uganda are largely inadequate, and the women face several barriers. Inadequacies include infrequent change of sanitary pads, lack of privacy during menstrual hygiene practices, and indiscriminate disposal of used sanitary products like used pads. On a positive front, bathing during menstruation with soap was frequent and not restricted. Barriers to menstrual hygiene management included unhygienic sanitary facilities, unreliable access to clean water, and inadequate sanitary facilities. These inadequacies should be the focus of prison health authorities.

The prison authorities should provide sufficient sanitary products like pads, and knickers including soap, construct more sanitary facilities, educate about the safe disposal of used sanitary products, and provide sufficient clean water to promote good menstrual hygiene management among incarcerated women. We, therefore, implore prison authorities to provide sufficient clean water supply to prevent water and sanitation hygiene-related diseases among incarcerated women at the Luzira Prison.

## Data Availability

All the dataset generated and/or analysed during the current study are included in this published article.

## List of abbreviations

CIU: Clarke International University
REC: Research Ethics Committee.
IDI: In-depth Interview
KII: Key Informant Interview.SD:Standard deviation.
